# Formation AgI and ZnI_2_ Nanocrystals in AgI-ZnI_2_-SiO_2_ Hybrid Powders

**DOI:** 10.3390/nano15241875

**Published:** 2025-12-13

**Authors:** Anastasiia Averkina, Igor Valtsifer, Vladimir Strelnikov, Natalia Kondrashova, Viktor Valtsifer

**Affiliations:** Institute of Technical Chemistry of Ural Branch of RAS—Branch of Perm Federal Research Centre of Ural Branch of RAS, 3 Academika Koroleva Str., 614068 Perm, Russia

**Keywords:** hybrids, silver iodide, artificial technology, silica dioxide

## Abstract

AgI and ZnI_2_ nanocrystals are key components for AgI-ZnI_2_-SiO_2_ hybrid powders (HPs), which could be potentially important for atmospheric artificial precipitation technology. HPs were created by the “Hydrothermal template cocondensation” method (“HTC” method). Mesoporous silica dioxide (MCM48, MCM41, SBA15, SBA16), silver iodides, and zinc iodides were simultaneously grown under specific conditions. The influence of silica dioxide on AgI and ZnI_2_ nanocrystals characteristics (phase, size, and thermal stability) were studied using various physicochemical analysis methods. In addition to crystal features, some structural and textural properties of the AgI-ZnI_2_-SiO_2_ hybrid as an individual agglomerate and its morphology were determined. This showed that nanocrystal features were dependent on synthesis condition. The influence of the nature of the reagent, which is pH-forming, was manifested at the initial stage of the process, and the morphology of the silica dioxide matrix controlled the crystal properties during the post-synthesis phase. It was established that the thermal stability of AgI and ZnI_2_ nanocrystals increased due to the protective shielding function of that SiO_2_ matrix.

## 1. Introduction

Artificial precipitation technology (cloud seeding) is a strategically important area for preventing hail, drought, and heavy rains. Cloud seeding is carried out by introducing special substances into clouds [1,2,3,4,5]. They broadly divide into hygroscopic (sodium, calcium, silver chlorides, etc.) and ice-forming (silver iodide, potassium iodide, dry ice (solid carbon dioxide), liquid propane) substances. When such compounds are dispersed into the atmosphere, the microphysical processes inside the cloud change and precipitation occurs.

The use of ice-forming substances has become the most widespread method of artificial precipitation technology. Among the abovementioned reagents, the most famous ice-forming substance is silver iodide [6,7,8,9,10]. The effectiveness of silver iodide is explained by its structural similarity: the molecular structure of silver iodide has the same hexagonal shape as natural ice. Thus, AgI acts as an “imitation” of natural ice crystals, which are needed to form snowflakes.

The study of the effect of silver iodide on the atmospheric moisture began at the end of the 20th century, so the scientific research on improving properties, and, consequently, improving the effectiveness of exposure, is still relevant [11,12,13]. Despite the high demand for silver iodide use in artificial precipitation technology, it has a number of disadvantages such as large crystal size, mobile polymorphism of the crystal lattice, and low thermal stability. In addition, silver iodide undergoes photolysis. These parameters are crucial because they make it difficult or impossible for crystalline silver iodide to interact with ice crystals [14,15,16,17,18,19,20].

The influence of these parameters is understandable, as there are some problems accompanying the process [6,7,18,21,22]. First, large crystals of natural AgI are not able to interact with ice crystals. Secondly, silver iodide is heated in the delivery vehicles and then is degraded to metallic silver and silver oxide [12,23,24]. The preserved silver iodide can undergo photolysis after spraying [25,26,27]. As a result, the amount of active silver iodide released into the atmosphere and capable of reacting with ice crystals is not more than 20–25 wt.% from the initial amount. Therefore, it is extremely necessary to maximize the creation and preservation of the active silver iodide amount. There are common approaches to eliminating these negative parameters.

The first approach is to reduce dispersion of silver iodide by changing the parameters of the crystals chemical synthesis. The approaches are based on the use of surfactants (or similar compounds) to stabilize and prevent the aggregation of growing crystals in the volume of solutions [28,29,30,31,32,33]. The second way is to prevent the destruction of AgI by adding iodine donors such as copper iodide, zinc iodide, magnesium iodide, or iodates of these metals [6,34,35]. According to the available data, atomic iodine obtained as the result of the sublimation of donor compounds will create excessive pressure and will contribute to a shift in the reaction of thermal decomposition of silver iodide towards the preservation of the initial compound.

Previously, the authors created an AgI-SiO_2_ hybrid powder. The main idea of their experimental work was to intercalate AgI crystals to a SiO_2_ matrix. The authors based the formation of silver iodide crystals on a chemical reaction between silver nitrate and potassium iodide. The synthesis methods significantly increased the thermal stability of silver iodide and reduced the dispersion of its crystals [36,37]. The use of silica dioxide (rather than native silicon or polymer matrices) as the matrix of silver iodide crystals seems appropriate, since this will significantly increase the biotolerance of silver iodide and, in general, the hybrid for the environment [38,39,40,41,42].

The work represents the next stage in studying the physico-chemical foundations of formation of an AgI-ZnI_2_-SiO_2_ inorganic composite and improving its performance (in terms of increasing the thermal stability of silver iodide) as a potential reagent for artificial precipitation technology. The creation of the expanded hybrid powder is justified, since this will further increase the thermal stability of silver iodide. The expanded hybrid powder is a type of powder where silver iodide crystals and iodine donor crystals are included. In addition, the addition of iodine donors in the silica dioxide matrix will also significantly reduce the number of iodine donors (up to 10–15 times) compared to a simple (mixed introduction of these compounds) into the reagent.

Thus, in order to achieve this goal, it is necessary to solve a number of tasks related to the establishment of methods for the introduction of iodine donors into the hybrid and the determination of synthesis conditions for the characteristic features of AgI and ZnI_2_ nanocrystals.

## 2. Methods and Materials

“Hydrothermal template co-condensation” method (“HTC” method) was the basic method used for AgI-ZnI_2_-SiO_2_ HPs synthesis. The “HTC” method conditions and parameters were established by the authors in the following studies [36,37]. This method allowed for the simultaneous synthesization of silicon dioxide, silver iodide (from silver nitrate), and zinc iodide (from zinc nitrate) in the presence of excessive potassium iodide. Different types of mesoporous silicon dioxide (such as MCM48, MCM41, SBA15, and SBA16) served as the base for the silver iodide and zinc iodide crystals. The ratio [Ag]/[Si] = 0.08 was optimal because the formation of crystalline silver iodide began in the composite using the “HTC” method. This fact has been established and experimentally confirmed many times in previous studies.

Some substances were used for the synthesis: tetraethoxysilane (TEOS) (98%, Sigma Aldrich (MI, USA)), silver nitrate (99%, Sigma Aldrich), zinc nitrate (99%, Sigma Aldrich), potassium iodide (97%, Sigma Aldrich), cetyltrimethylammonium bromide (CTAB) (98%, Sigma Aldrich), Pluronic P-123, (98%, Sigma-Aldrich), Pluronic F-127, (98%, Sigma-Aldrich),sodium hydroxide (98%, Sigma Aldrich), Ammonium hydroxide solution (28–30%, Sigma Aldrich), and hydrochloric acid (36%, Sigma Aldrich).

The conditions of AgI-ZnI_2_-MCM48 HPs synthesis were the following parameters: hydrothermal aging time was 48 h, hydrothermal aging temperature was 120 °C, and pH of the sol was 10–12. The ratio of [TEOS]/[CTAB] was 1:0.44, and the ratio of [Ag]/[Si] was 0.08.

The conditions of AgI-ZnI_2_-MCM41 HPs synthesis were the following parameters: hydrothermal aging time was 48 h, hydrothermal aging temperature was 100 °C, and pH of the sol was 10–12. The ratio of [TEOS]/[CTAB] was 1:0.20, and the ratio of [Ag]/[Si] was 0.08.

The conditions of AgI-ZnI_2_-SBA15 HPs synthesis were the following parameters: hydrothermal aging time was 24 h, hydrothermal aging temperature was 80 °C, and pH of the sol was 2–3. The ratio of [TEOS]/[Pluronic P-123] was 1:0.02, and the ratio of [Ag]/[Si] was 0.08.

The conditions of AgI-ZnI_2_-SBA16 HPs synthesis were the following parameters: hydrothermal aging time was 24 h, hydrothermal aging temperature was 80 °C, and pH of the sol was 2–3. The ratio of [TEOS]/[Pluronic F-127] was 1:0.02, and the ratio of [Ag]/[Si] was 0.08 [36,37].

The ratio [Ag]/[Zn] was 1.00 for new AgI-ZnI_2_-SiO_2_ HPs. This [Ag]/[Zn] ratio is not optimal. One of the main tasks of the authors is to increase the effectiveness of silver iodide as a reagent for artificial deposition technologies. The authors believe that this ratio may be acceptable for forming initial assumptions about the effect of iodine donors on the thermal stability of silver iodide and for obtaining the first ideas about the characteristics of synthesized HPs.

The first stage was the preparation of the mother liquid solution. The pH-formative agent and CTAB/Pluronic P-123/Pluronic F-127 were added first. Then, TEOS was introduced. After the silica dioxide precursor was introduced into the solution, silver nitrate and zinc nitrate were added consistently. Potassium iodide was introduced at the end. After a certain time, the mother liquid solution was placed into the vessel for hydrothermal synthesis. The hydrothermal synthesis conditions are described above.

Then HPs were washed and dried in a drying oven at a temperature of 110 °C.

The next step was the annealing stage. This was a post-synthetic treatment to remove the structure-forming agent (CTAB, Pluronic P-123, Pluronic F-127). It took place in a muffle furnace in the air atmosphere at 550 °C temperature for 5 h.

The synthesized samples were characterized by various physicochemical methods of analysis.

The structure of the metal iodide crystals and powder materials in general was identified by XRD (X-ray analysis). Diffraction patterns were recorded using the XRD-7000 diffractometer (Shimadzu, Kyoto, Japan) using CuKα radiation (λ_av_ = 1.54 Å). The scan ranges were 1.4–10.0 and 10.0–80.0 degrees with a step of 0.010–0.005 degrees; accumulation time ranged from 1.5 to 2.0 s. The diffraction patterns were identified using the JSPDS powder diffraction file. Repeated reflection analysis using the Williamson–Hall method was used to obtain the size of metal iodide nanocrystals.

The morphological features of the inorganic hybrid samples were examined using a FEI Quanta FEG650 scanning electron microscope (ThermoFisher Scientific, Breda, The Netherlands).

Transmission electron microscopy (TEM) analysis was performed using a JEOL JEM 2100 at a 200 kV acceleration voltage. The spherical aberration coefficient, Cs, is 0.5 mm. The spatial resolution of this instrument is 0.14 nm for lattice resolution and 0.19 nm for point-to-point resolution. The calibration of the magnification of the high-resolution transmission electron microscopy (HRTEM) and the determination of the device constant for the electron diffraction measurements were performed using a gold foil. The processing and analysis of the HRTEM images were performed using the Gatan Digital Micrograph (DM) version 3.9.3 software, which allowed us to calibrate images, to apply masks and filters, to perform fast Fourier transformations (FFT) of both the image as a whole and its selected areas, and to conduct measurements.

The structural and textural properties of the HPs were assessed by low-temperature nitrogen adsorption with an ASAP 2020 analyzer (Micromeritics, Norcross, GA, USA). For the analysis, the samples were vacuum degassed at 350 °C for three hours. The specific surface area (S_BET_) and total pore volume (V_tot_) were determined using the BET method. The pore size distribution was characterized after desorption isotherms by the BJH method, covering a range of 1.7–300.0 nm.

The content of silver iodide and zinc iodide was measured using atomic absorption spectroscopy (Thermo Fisher Scientific iCE 3500, based in the USA). The titration method was also indirectly used to quantify target substances.

The thermal behavior of HPs was studied using a TGA/DSC 1 device (METTLER-TOLEDO, Greifensee, Switzerland) in the air atmosphere at the heating rate of 10 °C/min in a temperature range of 25–1000 °C.

To study moisture absorption under static conditions, the powder material was saturated with moisture in a desiccator, where the relative humidity was maintained at approximately 90%.

HP activity was investigated under dynamic conditions. The climatic chamber specially was utilized to create an oversaturated wet area and facilitate its precipitation. This climatic chamber was developed by the Institute of Technical Chemistry of the Ural Branch of Russian Academy of Sciences. The chamber was a hermetically sealed thermally insulated cubic structure with a volume of 8 m^3^. It was equipped with an ultrasonic humidification system capable of producing 310 cm^3^/h, a device for spraying HPs, and laser sensors (CDR-10X optical position sensors). The temperature and humidity conditions were regulated and maintained by the air-conditioning system within the chamber. The experiments were conducted at the temperature of −5 °C. The duration of the destruction process was calculated by subtracting the time when the hybrid was introduced into the oversaturated wet area from the moment when it became transparent.

## 3. Results and Discussion

The authors developed several methods for hybrid synthesis, and those methods were used for AgI-ZnI_2_-SiO_2_ powder creation [36,37]. Those methods allowed for AgI-ZnI_2_-SiO_2_ formation from different silica dioxide matrixes: MCM48, MCM41, SBA15, and SBA16. The synthesized HPs showed different characteristic features (structural, textural, and exploitation properties, and thermal stability of nanocrystals) due to various silica dioxide types.

**Figure 1:**
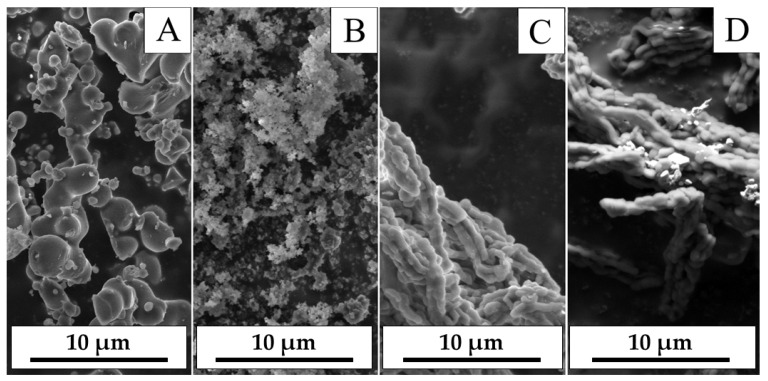
SEM-images of AgI-ZnI_2_-SiO_2_ HPs: (**A**)—AgI-ZnI_2_-MCM48, (**B**)—AgI-ZnI_2_-MCM41, (**C**)—AgI-ZnI_2_-SBA15, (**D**)—AgI-ZnI_2_-SBA16.

It was established that micro-agglomerate was a structural unit of powders, and the samples were polydispersed powders. This proved that the morphology of the HPs’s agglomerates was determined by the characteristics of the native silica dioxide matrix of the corresponding family [43,44]. SEM results show that AgI-ZnI_2_-MCM48 HP were worm-like agglomerates; AgI-ZnI_2_-MCM41 were grape-like agglomerates, and AgI-ZnI_2_-SBA15 and AgI-ZnI_2_-SBA16 were elongated spherical agglomerates (Figure 1). SiO_2_ type was confirmed by Small-Angle X-ray Scattering. The micro-agglomerate was the result of stable accumulation of single silica dioxide nanoparticles. It was assumed that electrostatic forces held silica dioxide nanoparticles together. The silver iodide and zinc iodide nanocrystals did not prevent self-assembly of powder micro-agglomerates.

**Figure 2:**
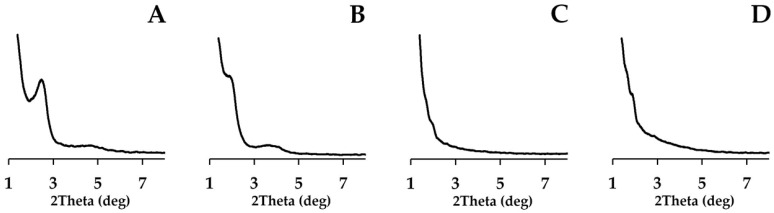
X-ray image of AgI-ZnI_2_-SiO_2_ HPs in small angles: (**A**)—AgI-ZnI_2_-MCM48, (**B**)—AgI-ZnI_2_-MCM41, (**C**)—AgI-ZnI_2_-SBA15, (**D**)—AgI-ZnI_2_-SBA16.

The X-ray phase analysis at low diffraction angles of 1–10° showed that silica dioxide was mesoporous silica dioxide of MCM and SBA families (Figure 2) [43,44]. The obtained X-ray images confirmed that different pore architectures appeared in the synthesized HPs. It was found that the synthesized samples on the SBA family matrix possess larger pores and significantly thicker walls. HPs on the MCM family have a very high specific surface area.

The SEM and X-ray results confirm that silver iodide and zinc iodide did not affect the structure of the silica dioxide matrix.

The low-temperature nitrogen sorption was used to assess the structural properties of HPs. It was established that a significant decrease in the specific surface area for the synthesized samples compared to the unmodified silica oxide matrix occurred (Table 1). The decrease was up to 20–80 times for this parameter. The results for the unmodified mesoporous silica dioxides are described in the scientific works by [45,46,47,48]. The changes in structural properties occurred for all the synthesized AgI-ZnI_2_-SiO_2_ HPs samples. It was found that the pore volume indicators and their linear dimensions significantly changed, as well as the specific surface area. This could be because silver iodide and zinc iodide nanocrystals were embedded into the surface, and they were the guests with respect to silica dioxide at the stage of the sol existence.

**Figure 3:**
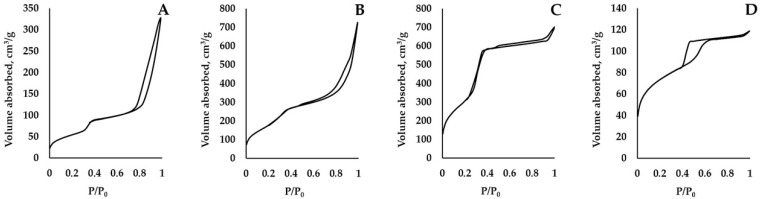
The AgI-ZnI_2_-SiO_2_ HP sorption isotherms: (**A**)—AgI-ZnI_2_-MCM48, (**B**)—AgI-ZnI_2_-MCM41, (**C**)—AgI-ZnI_2_-SBA15, (**D**)—AgI-ZnI_2_-SBA16.

**Figure 4:**
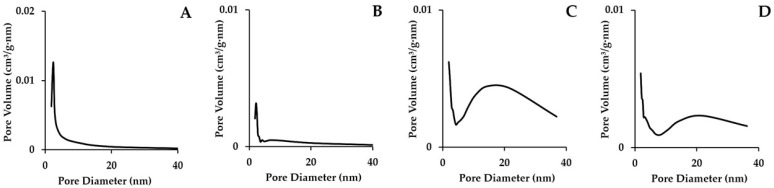
The pore size distribution curve for AgI-ZnI_2_-SiO_2_ HP samples: (**A**)—AgI-ZnI_2_-MCM48, (**B**)—AgI-ZnI_2_-MCM41, (**C**)—AgI-ZnI_2_-SBA15, (**D**)—AgI-ZnI_2_-SBA16.

We present the HP sorption isotherms images in Figure 3. Those isotherms were IV type isotherms (according IUPAC classification). Such types of isotherms described the micro- and mesopores in the HPs samples. The pore sizes of such samples are usually 2–50 nanometers (the data is confirmed by information in Table 1). The hysteresis loop (Figure 3) indicates the phenomenon of capillary condensation inside the pores. The results of low-temperature nitrogen sorption also show that the pore size distribution is narrow (Figure 4).

**Figure 5:**
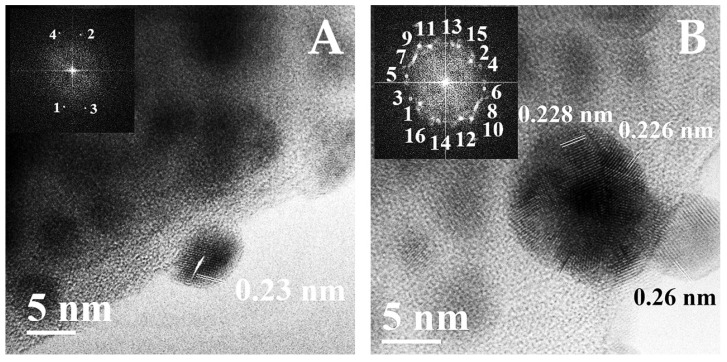
TEM-images of AgI-ZnI_2_-MCM48 HP: (**A**)—ZnI_2_ particle at 500,000× magnification in high (atomic) resolution mode, (**B**)—AgI particle at 600,000× magnification in high (atomic)-resolution mode.

The changes in structural and textural properties might be a consequence of the fact that crystals of silver iodide and zinc iodide were intercalated on the irregularities (natural bends of the microagglomerate) of the relief and in the pores and the interporous space, as well as into volumetric layers of the silica dioxide matrix. This was evidenced by the results of the TEM (Figure 5). Figure 5A shows a transmission electron microscope image (TEM) of the AgI-ZnI_2_-MCM48 sample obtained at 500,000× magnification in high (atomic)-resolution mode. Dark spots in the central part of the image corresponded to crystalline particles in the amorphous matrix, in which electron beam scattering occurred. A particle with crystal lattice fringes is visible in the lower part of the image. The fast Fourier transform (FFT) is shown in the inset in the upper right part of Figure 5A. Decoding points 1–2 and 3–4 in the FFT yielded interplanar spacings of 0.23 and 0.21 nm. These interplanar spacings corresponded to the tetragonal structure of ZnI_2_ (JCPDS PDF 01-070-1224). Figure 5B shows a transmission electron microscope image of the AgI-ZnI_2_-MCM48 sample obtained at 600,000× magnification in high (atomic)-resolution mode. Two spherical particles are visible in the central part of the image. On the right, a small particle slightly larger than 7 nm has an interplanar spacing of 0.265 nm, which corresponds to the tetragonal structure of AgI (*Miersite*) (JCPDS PDF 00-022-1122). The larger particle consists of seven AgI nanocrystallites. The fast Fourier transform (FFT) is shown in the inset in the upper left part of Figure 5B. Decoding points 1–2, 3–4, 5–6, and 7–8 in the FFT yielded interplanar spacings of 0.22, 0.27 nm, 0.20, and 0.13 nm. These interplanar distance values corresponded to the hexagonal structure of AgI (*Iodargyrite*) (JCPDS PDF 09-003-0940). A similar picture was observed for the remaining hybrid samples.

**Figure 6:**
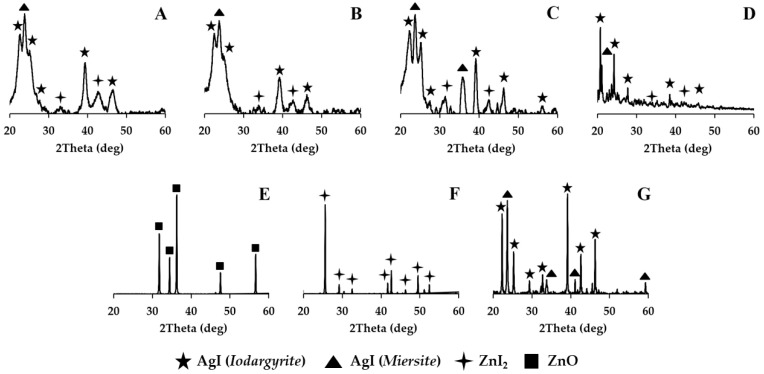
X-ray image of samples: (**A**)—AgI-ZnI_2_-MCM48 HP, (**B**)—AgI-ZnI_2_-MCM41 HP, (**C**)—AgI-ZnI_2_-SBA15 HP, (**D**)—AgI-ZnI_2_-SBA16 HP, (**E**)—ZnO, (**F**)—ZnI_2_, (**G**)—AgI.

The characteristic features of AgI and ZnI_2_ nanocrystals were defined using an X-ray. The average sizes of metal iodide crystallites obtained by calculation according to the Williamson–Hall method are presented in Table 2 [49,50]. It was found that silver iodide nanocrystals (in the crystalline phases of *Miersite*, *Iodargyrite*) and zinc iodide nanocrystals were formed in all the synthesized HPs under the selected synthesis conditions. It also shown that all HP samples did not contain zinc oxide. This fact was established according to the X-ray results. It was evidenced by the characteristic peaks in the X-ray diffraction patterns (Figure 6). The type of silica dioxide matrix had no impact on the likelihood of silver iodide and zinc iodide nanocrystal formation. However, a notable difference was observed. The size of the crystallites increased in the sequence SBA16, SBA15, MCM41, and MCM48.

The X-ray results show (Figure 6) that AgI and ZnI_2_ nanocrystals were created in HPs, because the X-ray images for these samples have specific peaks. The formation of all types of nanocrystals was for all AgI-ZnI_2_-SiO_2_ hybrids. The X-ray images of pure substances (Figure 6E–G) also confirmed the formation of crystals in the HPs. There are overlaps of characteristic peaks for silver iodide and zinc iodide noted [51,52,53,54]. This phenomenon is typical for closely related compounds.

The correlation between the linear dimensions and the type of mesoporous silica dioxide could be explained by certain principles.

First and foremost, the influence of the pore size within the silica matrix (and, consequently, the interpore space) became evident. It is well-known that the MCM family has smaller pores than the SBA family. Secondly, the influence of the pH-forming agent was manifested. The pH-forming agent determined the activity of the medium at the stage of preparation of the mother liquid solution and at the stage of hydrothermal exposure. Specifically, NaOH was used in the synthesis of MCM48 particles, while NH_4_OH was employed for MCM41. On the other hand, HCl was utilized for SBA16 and SBA15 particles. Potentially, the influence of the pH-forming agent could be manifested through the different solubility of silver intermediates at the stage of preparation of the mother liquid solution and at the stage of hydrothermal exposure. Intermediates could occur because pH-forming agents could also form complex or poorly soluble compounds with silver cations. The problem of influence is a discursive direction.

The influence of the listed substances on the reaction pathway and the intensity between silver nitrate, zinc nitrate, and potassium iodide varied significantly. These substances altered the pH of the solution and affected the kinetic parameters of silver iodide and zinc iodide formation. This effect was due to the ability of silver and zinc cations (zinc compounds are ampholytic substances in addition) to form either sparingly soluble compounds (such as silver oxide, zinc hydroxide, silver chloride, etc.) or complex compounds. Consequently, potassium iodide indirectly interacted with silver nitrate and zinc nitrate. This hypothesis will be further investigated.

**Figure 7:**
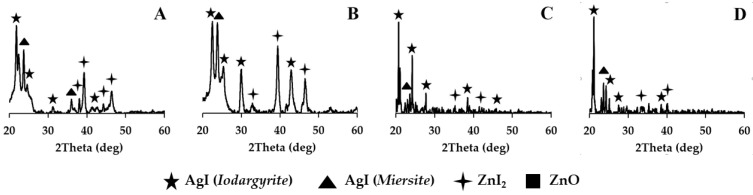
X-ray image of AgI-ZnI_2_-SiO_2_ HPs after high-temperature exposure: (**A**)—AgI-ZnI_2_-MCM48, (**B**)—AgI-ZnI_2_-MCM41, (**C**)—AgI-ZnI_2_-SBA15, (**D**)—AgI-ZnI_2_-SBA16.

**Figure 8:**
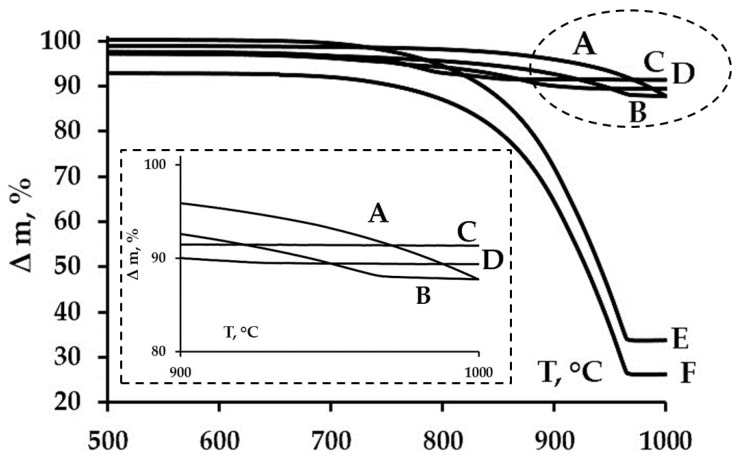
The thermal gravimetric curves of samples: A—AgI-ZnI_2_-MCM48 HP, B—AgI-ZnI_2_-MCM41 HP, C—AgI-ZnI_2_-SBA15 HP, D—AgI-ZnI_2_-SBA16 HP, E—AgI, F—ZnI_2_.

**Figure 9:**
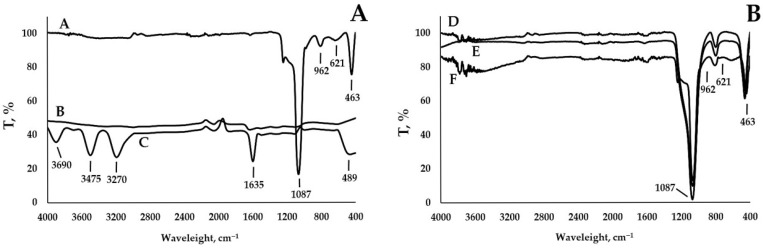
The thermal gravimetric curves of samples: (**A**) A—AgI, B—ZnI_2_, C—MCM48, (**B**) D—AgI-MCM48 HP, E—ZnI_2_-MCM48 HP, F—AgI-ZnI_2_-MCM48 HP.

The AgI thermal stability was an important feature of the AgI-ZnI_2_-SiO_2_ reagent. AgI’s thermal stability was more significant than ZnI_2_’s thermal stability, because ZnI_2_ will also be destroyed under the influence of high temperatures. However, thermal destruction of zinc iodide is important because it helps to prevent the sublimation of silver iodide, and iodine donors can form in the local combustion zone. AgI’s thermal stability was studied using an X-ray (Figure 7), thermal gravimetric analysis (Figure 8), differential scanning calorimetry (Figure 9), and atomic absorption spectroscopy with titration (Table 3). AgI’s thermal stability preserved AgI crystals at high-temperature exposure at 1000 °C for 10 min in the author’s experiments. Of course, the selected mode did not fully reflect the combustion temperature of the delivery vehicle. Thus, the authors believe that the conditions for studying thermal stability were adequate, and the experimental results were reliable.

The hybrid samples underwent high-temperature treatment. The X-ray analysis of these powders revealed that both silver iodide and zinc iodide remained intact in their composition. Zinc oxide was not detected in all of the powders. The presence of silver iodide and zinc iodide crystals was consistently observed in all hybrid samples, irrespective of the type of silica dioxide matrix (Figure 7). This was confirmed by the characteristic peaks seen in the X-ray diffraction patterns.

Thermal stability was assessed using the thermal gravimetric analysis, which revealed the dynamics of silver iodide and zinc iodide decomposition under high-temperature conditions. There were not any qualitative or quantitative changes in the silica dioxide matrix detected during the heating procedure. It was particularly challenging to disentangle the thermal decomposition processes of silver iodide and zinc iodide. The authors conducted experiments where AgI-SiO_2_ and ZnI_2_-SiO_2_ hybrids, utilizing MCM48, MCM41, SBA15, and SBA16 as silica dioxide matrices, were separately exposed to high temperatures. However, these experiments did not provide a clear single influence. It was observed and confirmed that the delayed sublimation zone expansion (Figure 8) occurred at the temperatures ranging from 750 to 900 °C for AgI-ZnI_2_-SiO_2_ hybrids, compared to pure silver iodide and zinc iodide (Figure 8, curve E, F). Zinc iodide and silver iodide passed their melting temperatures in the ranges of 440–450 °C and 550–560 °C, respectively [55,56,57,58,59]. But there was a significant weight loss of samples for these compounds that began after 700 °C. It was noted that the character of thermal decomposition of silver iodide and zinc iodide were similar. However, all other things being equal, the degradation of zinc iodide was more pronounced. This criterion was a positive factor, since zinc iodide was introduced as a donor to stabilize silver iodide under high-temperature exposure.

DSC analysis was performed for all hybrid powder materials and pure individual components (Figure 9). The DSC-curve A described the thermal behavior of pure silver iodide. The DSC-curve A exhibited peaks characterizing the phase transition (146 °C) and melting of the crystal lattice (558 °C). The DSC-curve B describes the thermal behavior of pure zinc iodide. The DSC-curve B contains a peak that describes the melting of the crystal lattice (446 °C). The DSC-curve C describes the thermal behavior of the original mesoporous silica MCM48. No extreme points are observed on the DSC-curve C. The DSC-curves D, E, and F describe the thermal behavior of HPs with one or two metal iodide components. It is noted that there were no peaks of phase transitions and melting on these curves lack characteristic peaks. Minor disturbances were observed in regions above 700 °C. This was due to the sublimation of iodine from the metal iodides. This phenomenon was consistent with thermogravimetric analysis data.

Table 3 shows the results of the determination of the quantity (wt. %) of composite components after the high-temperature exposure. The amount of silver iodide was determined based on the results of the X-ray diffraction (according to the built-in function of the device). The amount of zinc iodide and zinc oxide was calculated using atomic absorption spectroscopy in combination with complex metric titration [60,61,62], since zinc oxide was X-ray amorphous and was not determined by X-ray phase analysis. The amount of silver oxide was not determined, since the objective was to determine the amount of silver iodide in the HPs. It was found that the total content of zinc compounds was approximately comparable to the content of silver compounds, which was consistent with the data on the composition of the hybrid powder.

It was found that the type of silica dioxide matrix changed the thermal stability of silver iodide and zinc iodide. The data that was obtained through this method confirmed the protective screening property of the silica dioxide matrix. It was also determined that the quantity of preserved silver iodide and zinc iodide increased in the series SBA15, SBA16, MCM41, and MCM48. The amount of silver iodide in the hybrid, the one that was preserved, was sufficient because if pure silver iodide was exposed to the high temperature then small amount of AgI (25–30 wt. %) was saved. This phenomenon can be attributed to the silica dioxide matrix providing protection of the silver iodide and zinc iodide nanocrystals. MCM48 exhibited stronger protective properties compared to the SBA15 silica dioxide due to its more complex surface structure, which is characterized by high structural and textural indicators.

Some of the existing exploitation characteristics were moisture absorption under static conditions and the dynamics of atmospheric moisture condensation. The results of the study are presented in Table 4. We are going to conduct field tests as the next step after the laboratory tests.

The analysis of potential operational characteristics (Table 4) revealed the fact that the synthesized hybrids exhibited at least two distinct mechanisms affecting the atmospheric moisture. The first mechanism involved condensation due to the silica dioxide matrix, as evidenced by moisture absorption under static conditions. The second mechanism was the generation of descending air flows, as indicated by the precipitation dynamics. It was observed that the condensation activity in relation to atmospheric moisture increased in the sequence MCM41, MCM48, SBA16, and SBA15. This trend was linked to the porosity of the silica matrix, with the pore size playing a more significant role than the surface area. Consequently, the condensation efficiency rose in proportion with the increase in mesoporous silica dioxide pore space. The intensity of the generation descending air flows was comparable across all the types of AgI-ZnI_2_-SiO_2_ HPs. The duration of destruction for AgI-ZnI_2_-MCM41 was the longest, because MCM41 micro-agglomerates might create a stable suspension in atmospheric air.

## 4. Conclusions

The hydrothermal template co-condensation method synthesized the hybrid materials with AgI and ZnI_2_ nanocrystals within mesoporous silica dioxide matrices (MCM48, MCM41, SBA15, SBA16). The hybrids, that were formed had well-defined mesoporous silica dioxide structures with metal halide nanocrystals which were integrated into the matrix. Listed crystals might be persist at the silica surface via weak physical interactions. The selected [Ag]/[Zn] can be the starting point for further iteration in order to determine the optimal ratio of [Ag]/[Zn].

It was established that the characterization of the silica dioxide matrix has been shown to significantly influence the morphological attributes of AgI-ZnI_2_-SiO_2_ HPs.

The process of silver iodide and zinc iodide nanocrystals intercalation onto the SiO_2_ matrix systematically reduced the structural–textural characteristics. The reductions varied in the matrix type: 40 m^2^/g for MCM48, 33 m^2^/g for MCM41, 27 m^2^/g for SBA15, and 25 m^2^/g for SBA16. The deposition also altered the pore volume metrics and the linear dimensions across all the matrix types.

The structure of mesoporous silicon dioxide did not have a direct impact on the growth or morphology of silver iodide and zinc iodide nanocrystals. However, the properties of the matrices and pH-forming substances used in the synthesis indirectly influenced the crystal formation.

The morphology and physico-chemical properties of mesoporous silicon dioxide significantly influenced the final morphology and the sizes of metal halide nanocrystals.

The silica dioxide matrix and zinc iodide significantly improved the thermal stability of silver iodides due to their protective shielding effect and the creation of excess iodine. The exposure to high-temperature showed a different content of silver iodide in various mesoporous silica matrices: 71% for MCM48, 68% for MCM41, 66% for SBA15, and 64% for SBA16.

The analysis of the AgI-ZnI_2_-SiO_2_ characteristics revealed the differences that formed the theoretical basis for the new reagents’ classification for artificial humidification. These differences can optimize their use under various atmospheric conditions, including cloud moisture content and altitude above the sea level. This approach will improve the accuracy of atmospheric design and expand the possibilities of reagent use in various environmental conditions.

The synthesized reagent will significantly enhance the possibility of weather control with the help of artificial technologies. It will optimize atmospheric dynamics, improving the management of dangerous weather.

## Figures and Tables

**Table 1:** The structural and textural properties of AgI-ZnI_2_-SiO_2_ HPs.

HP Sample	Surface Area, S_BET_, m^2^/g	Total Volume, cm^3^/g	Pore Diameter, nm (Desorption)
AgI-ZnI_2_-MCM48	40	0.07	6.77
AgI-ZnI_2_-MCM41	33	0.09	7.12
AgI-ZnI_2_-SBA15	27	0.11	8.96
AgI-ZnI_2_-SBA16	25	0.10	9.28
MCM48	1635	0.97	2.6
MCM41	1088	0.84	3.2
SBA15	688	0.72	3.9
SBA16	617	0.67	4.6

**Table 2:** The average crystallite size in the AgI-ZnI_2_-SiO_2_ HPs.

HP Sample	AgI-Crystaline Size, nm	ZnI_2_-Crystaline Size, nm
AgI-ZnI_2_-MCM48	35	27
AgI-ZnI_2_-MCM41	29	23
AgI-ZnI_2_-SBA15	25	21
AgI-ZnI_2_-SBA16	27	19

**Table 3:** The average content of components in the AgI-ZnI_2_-SiO_2_ HPs after high-temperature exposure (wt. %).

HP Sample	AgI	ZnI_2_	ZnO
AgI-ZnI_2_-MCM48	71	9	57
AgI-ZnI_2_-MCM41	68	7	60
AgI-ZnI_2_-SBA15	66	6	64
AgI-ZnI_2_-SBA16	64	6	67

**Table 4:** The moisture absorption under the static and dynamics conditions for the AgI-ZnI_2_-SiO_2_ HPs.

HP Sample	Moisture Absorption, wt. %	The Duration of Destruction, s
AgI-ZnI_2_-MCM48	32	92
AgI-ZnI_2_-MCM41	29	105
AgI-ZnI_2_-SBA15	26	82
AgI-ZnI_2_-SBA16	27	76

## Data Availability

The original contributions presented in this study are included in the article/Supplementary Materials. Further inquiries can be directed to the corresponding authors.

## Supplementary Materials

The following supporting information can be downloaded at: https://www.mdpi.com/article/10.3390/nano15241875/s1, Figure S1. IR spectra of samples: A—MCM48, B—AgI, C—ZnI2, D—AgI-MCM48, E—ZnI2-MCM48, F—AgI-ZnI2-MCM48. Refs. [33,63] are cited in the supplementary material file.
