# Supplementary material for: Formation AgI and ZnI2 Nanocrystals in AgI-ZnI2-SiO2 Hybrid Powders
Source: Nanomaterials (Basel). 2025 Dec 13;15(24):1875. doi: 10.3390/nano15241875 (PMC12736330; doi:10.3390/nano15241875)
Supplement: Supplementary file 1 [file nanomaterials-15-01875-s001.zip › nanomaterials-4001953-supplementary.pdf]

Supplementary Materials

IR spectra were obtained for all HPs, as well as for individual components (Figure S1). The authors presented the results for a series of samples synthesized on MCM48. The results of hybrids containing one type of metal iodide were also presented for comparison. The results for the remaining samples were similar. The identification of spectra was carried out using available public information [33,63] and reference materials SDBS.

ZnI_2 sample (Figure S1 curve C): The presence of a wide diffuse peak in the range 3000–3800 cm^{-1} can be explained by overlapping of the following bands: stretching vibration of hydroxyl; stretching vibrations of adsorbed water molecules. A peak in the range of 1640–1600 cm^{-1} is a very common and strong signal for water. The appearance of peaks characteristic of water molecules in a zinc iodide sample is understandable, since zinc iodide can absorb air moisture. The fundamental Zn-I stretching vibrations occur at much lower wavenumbers, typically in the far-infrared region around 400–550 cm^{-1}.

AgI sample (Figure S1 curve B): It was confirmed that the pure silver iodide has no characteristic absorption peaks in the standard FTIR at a range from 4000 cm^{-1} to 400 cm^{-1}.

MCM48 sample (Figure S1 curve A): In the FTIR spectrum, the region around 1084–1090 cm^{-1} is dominated by a very strong, broad absorption band corresponding to the asymmetric stretching vibrations of the bridging Si-O-Si bond. The stretching vibrations of surface hydroxyl groups of Si-OH appear at 960–964 cm^{-1}. The most prominent Si-O peaks are located at approximately 460 cm^{-1}. The weak peak of about 610–620 cm^{-1} can be attributed to the stretching modes of the Si-Si bond. A peak in the range of 950–960 cm^{-1} is characteristic of the stretching vibration of a non-bridging silicon-oxygen bond (Si-O-), often found in silanol groups (Si-OH).

AgI-MCM48, ZnI_2-MCM48, and AgI-ZnI_2-MCM48 samples (Figure S1 curves D–F): It was found that the peaks in the IR spectrum were similar to the peaks for pure MCM48. No additional peaks were detected. The data obtained suggest that no chemical bond "Ag-O-Si" was formed.

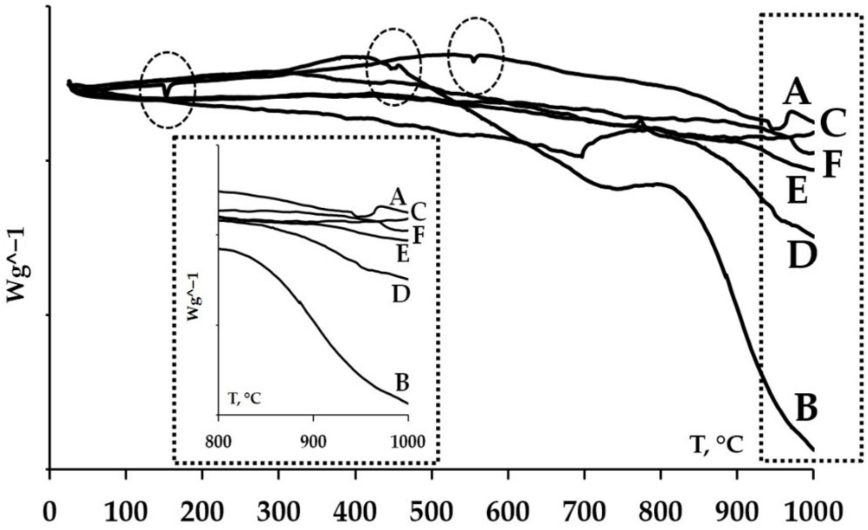

Figure S1. IR spectra of samples: A—MCM48, B—AgI, C—ZnI2, D—AgI-MCM48, E—ZnI2-MCM48, F—AgI-ZnI2-MCM48.
